# The value of contrast-enhanced ultrasound in predicting postoperative recurrence of hepatocellular carcinoma: A protocol for systematic review and meta-analysis

**DOI:** 10.1097/MD.0000000000025984

**Published:** 2021-06-04

**Authors:** Jieying Fu, Jia Tang, Huan Luo, Wencui Wu

**Affiliations:** a Department of Ultrasound Medicine, The Second Affiliated Hospital of Hainan Medical College, Haikou, Hainan Province; b Department of Ultrasound Medicine, The Ninth People's Hospital of Chongqing, Chongqing; c Department of Ultrasound Medicine, Haikou Hospital of the Maternal and Child Health, Haikou, Hainan Province, China.

**Keywords:** contrast-enhanced ultrasound, diagnosis, hepatocellular carcinoma, meta-analysis, protocol, recurrence

## Abstract

**Background:**

As one of the key factors, postoperative recurrence of hepatocellular carcinoma (HCC) influences the therapeutic effects and survival period of patients. Therefore, the early diagnosis of postoperative recurrence of HCC plays an important role in improving the therapeutic effects and prognosis. Contrast-enhanced ultrasound (CEUS) plays an important role in the early diagnosis of postoperative recurrence of HCC. However, the accuracy of CEUS in predicting postoperative recurrence of HCC is still controversial. Therefore, in this study, a meta-analysis was carried out to further evaluate the accuracy of CEUS in predicting postoperative recurrence of HCC, thus providing evidence support for the early diagnosis of HCC.

**Methods:**

The literatures on the CEUS diagnosis of postoperative recurrence of HCC were collected by searching China National Knowledge Infrastructure, Wanfang, China Biology Medicine disc, PubMed, EMBASE, Cochrane Library, and Web of Science on computer. The retrieval time is set from the start of the database until April 2021. The meta-analysis of the literatures that meet the quality standards was conducted by Stata 16.0 software.

**Results:**

The results of this meta-analysis will be submitted to a peer-reviewed journal for publication.

**Conclusion:**

This study will provide evidence support for the accuracy of CEUS in the diagnosis of postoperative recurrence of HCC.

**Ethics and dissemination:**

The private information from individuals will not be published. This systematic review also should not damage participants’ rights. Ethical approval is not available. The results may be published in a peer-reviewed journal or disseminated in relevant conferences.

**OSF Registration Number:**

DOI 10.17605/OSF.IO/HB46W.

## 1 Introduction

Hepatocellular carcinoma (HCC) is one of the most common cancers in China.^[1,2]^ According to reports, the mortality rate ranks the third among malignant tumors in digestive system.^[3]^ In recent years, with the development of economy and the change of people's living habits, the incidence and mortality of the disease are increasing year by year. Postoperative recurrence of HCC is one of the key factors affecting the therapeutic effects and survival period of patients.^[4]^ Therefore, the early diagnosis of postoperative recurrence of HCC plays an important role in improving the therapeutic effects and prognosis.

At present, the clinical methods adopted to determine the recurrence of HCC include AFP detection and imaging examination.^[5,6]^ The former is simple to be operated and is widely used in clinic, with high value in the diagnosis of HCC.^[7]^ The latter includes ordinary ultrasound, contrast-enhanced ultrasound (CEUS), computed tomography (CT), magnetic resonance imaging, and so on.^[8–10]^ In recent years, with the continuous improvement of CEUS technology and the application of ultrasound contrast agent, it displays more and more obvious advantages in the diagnosis of liver space-occupying lesions.^[11]^

The diagnostic performance of CEUS for postoperative recurrence of HCC has been continuously evaluated and confirmed as well. However, there exist inconsistencies in the effects in various literatures.^[12–18]^ The purpose of this study is to perform meta-analysis to explore the value of CEUS in predicting the postoperative recurrence of HCC, thus providing reference basis for early clinical diagnosis.

## 2 Methods

### 2.1 Study registration

The protocol of the systematic review has been registered on Open Science Framework (registration number: DOI 10.17605/OSF.IO/HB46W). It was reported by following the guideline of preferred reporting items for systematic reviews and meta-analysis protocol statement.^[19]^

### 2.2 Inclusion criteria for study selection

Inclusion criteria:

(1) To explore the diagnostic value of CEUS in the diagnosis of postoperative recurrence of HCC.
(2) All patients who accepted the operation of HCC were included.
(3) The gold standard was clinicopathological diagnosis, comprehensive imaging examination (enhanced CT, magnetic resonance imaging), and long-term clinical follow-up results.
(4) The data of the 4 tables of diagnostic tests can be obtained directly or indirectly.

Exclusion criteria:

(1) Literatures with incorrect data sources, incomplete data, and incorrect statistical methods.
(2) Literatures with repeated research data.
(3) Case reports, reviews, cell, or animal studies.

### 2.3 Data sources and search strategy

This study conducted a literature search in the China National Knowledge Infrastructure, Wanfang, China Biology Medicine disc, PubMed, EMBASE, Cochrane Library, and Web of Science. The retrieval time is set from the build of the database until April 2021. The language restrictions are Chinese and English. The search strategy for PubMed is displayed in Table 1.

**Table 1:** PubMed search strategy.

Number	Search terms
#1	Carcinoma, Hepatocellular[MeSH]
#2	Hepatocellular Carcinoma[Title/Abstract]
#3	Hepatoma[Title/Abstract]
#4	Liver Cancer, Adult[Title/Abstract]
#5	Liver Cell Carcinoma[Title/Abstract]
#6	Liver Cell Carcinoma, Adult[Title/Abstract]
#7	Adult Liver Cancer[Title/Abstract]
#8	Adult Liver Cancers[Title/Abstract]
#9	Cancer, Adult Liver[Title/Abstract]
#10	Cancers, Adult Liver[Title/Abstract]
#11	Carcinoma, Liver Cell[Title/Abstract]
#12	Carcinomas, Hepatocellular[Title/Abstract]
#13	Carcinomas, Liver Cell[Title/Abstract]
#14	Cell Carcinoma, Liver[Title/Abstract]
#15	Cell Carcinomas, Liver[Title/Abstract]
#16	Hepatocellular Carcinomas[Title/Abstract]
#17	Hepatomas[Title/Abstract]
#18	Liver Cancers, Adult[Title/Abstract]
#19	Liver Cell Carcinomas[Title/Abstract]
#20	or/1-19
#21	Recurrence[MeSH]
#22	Recrudescence[Title/Abstract]
#23	Relapse[Title/Abstract]
#24	Recrudescences[Title/Abstract]
#25	Recurrences[Title/Abstract]
#26	Relapses[Title/Abstract]
#27	Neoplasm Recurrence, Local[MeSH]
#28	Local Neoplasm Recurrence[Title/Abstract]
#29	Local Neoplasm Recurrences[Title/Abstract]
#30	Locoregional Neoplasm Recurrence[Title/Abstract]
#31	Neoplasm Recurrence, Locoregional[Title/Abstract]
#32	Neoplasm Recurrences, Local[Title/Abstract]
#33	Recurrence, Local Neoplasm[Title/Abstract]
#34	Recurrence, Locoregional Neoplasm[Title/Abstract]
#35	Recurrences, Local Neoplasm[Title/Abstract]
#36	Locoregional Neoplasm Recurrences[Title/Abstract]
#37	Neoplasm Recurrences, Locoregional[Title/Abstract]
#38	Recurrences, Locoregional Neoplasm[Title/Abstract]
#39	or/21-38
#40	Contrast-enhanced ultrasound[Title/Abstract]
#41	CEUS[Title/Abstract]
#42	or/40-41
#43	Diagnos∗[Title/Abstract]
#44	Sensitivity[Title/Abstract]
#45	Specificity[Title/Abstract]
#46	ROC curve[Title/Abstract]
#47	or/43-46
#48	#20 and #39 and #42 and #47

### 2.4 Data collection and analysis

#### 2.4.1 Study selection

**Figure 1:**
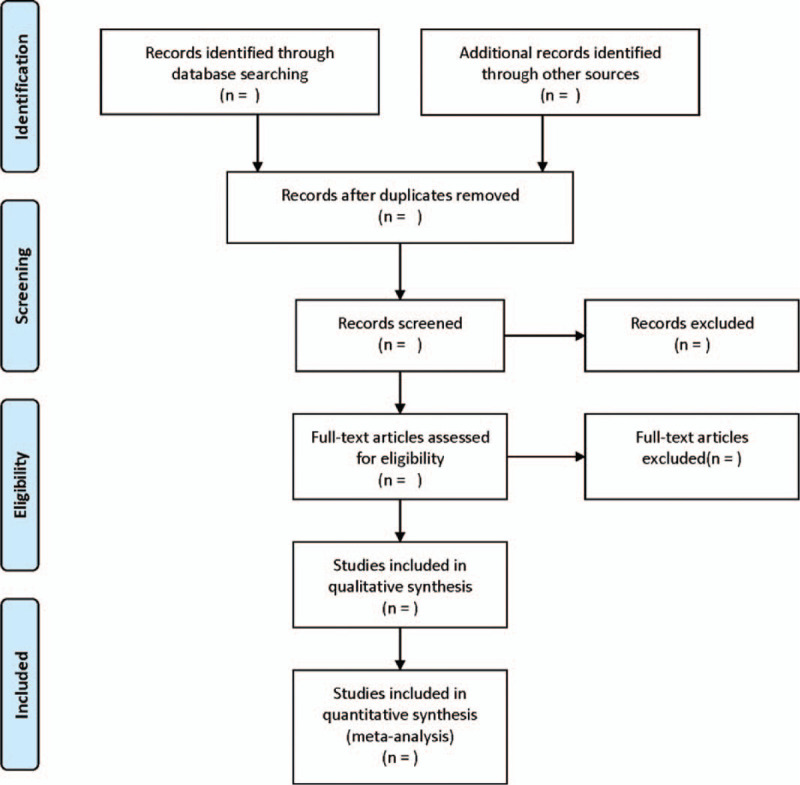
Flow diagram of literature retrieval.

All literatures were screened independently by 2 researchers based on the inclusion and exclusion criteria. In case of disagreement, a decision is made through discussion or consultation with relevant experts. The screening flow chart of this study is demonstrated in Figure 1.

#### 2.4.2 Data extraction

The following information was extracted: first author, year of publication, type of study, language, sample size, average age, gold standard, as well as true positive, false positive, true negative, and false negative. When the data collected by the 2 researchers are inappropriate, an agreement is reached through consultation. If the disagreement still exists after consultation, a third party (not the researcher) should be asked to make a decision.

#### 2.4.3 Dealing with missing data

If there are insufficient or missing data in the literature, the authors will be contacted via email. If the data are still not available, only the current available data will be analyzed and the potential impacts will be discussed.

### 2.5 Quality assessment

The methodological quality of the included studies was assessed by following quality assessment of diagnostic accuracy studies-2 criteria,^[20]^ including 11 evaluation criteria. Each standard is evaluated by “yes,” “no,” and “unclear.” “Yes” refers to that it meets this standard, “No” means that it does not meet this standard or is not mentioned, and “unclear” is partially consistent or unable to obtain sufficient information for evaluation. If the 11 standards are met, the quality of the literature will be rated as “A;” if more than 1 item is “unclear,” it will be rated as “B;” if “no” occurs, it will be rated as “C.”

### 2.6 Statistical analysis

All of the above statistical analyses were performed with Stata 16.0 (StataCorp LLC, college station, TX). We calculated pooled sensitivity (SEN), specificity, positive likelihood ratio, negative likelihood ratio, diagnostic odds ratio, area under the curve, and their 95% confidence interval. What's more, the pooled diagnostic value of CEUS through the summary receiver operating characteristic curve, and area under the curve was tested. The threshold effects were detected by applying spearman correlation coefficient. The calculation of heterogeneity was caused by the nonthreshold effects of Cochrane-*Q* and *I*^2^ values. Meanwhile, a fixed effect model (without obvious inhomogeneity) or a random effect model (with significant heterogeneity) was employed to merge the data. The statistical test level was α = 0.05.

### 2.7 Subgroup analysis

A subgroup analysis will be made on the basis of gold standard type, study type, surgery type, and threshold.

### 2.8 SEN analysis

We will adopt the one-by-one exclusion method to analyze the SEN of the results.

### 2.9 Reporting bias

The publication bias was determined by conducting Deeks’ funnel plot asymmetry test.

### 2.10 Ethics and dissemination

Since the program does not include the recruitment of patients and the collection of personal information, it does not require the approval of the Ethics Committee.

## 3 Discussion

The postoperative recurrence of HCC is high.^[21]^ At present, ultrasound and enhanced CT are routinely used for regular follow-up. CEUS technology is also applied more and more widely. The neovascularization of recurrent and metastatic foci after HCC is abundant, and it is mainly supplied by hepatic artery.^[22]^ When the contrast medium reached the hepatic artery, the lesion developed immediately, the intensity was higher than that of the surrounding normal liver tissue, homogeneity or inhomogeneity is enhanced, and the portal vein phase disappeared rapidly. Under the background of liver cirrhosis, CEUS can judge HCC and atypical hyperplastic nodules by contrast-enhanced imaging,^[23]^ because CEUS can observe every moment from the development of the hepatic artery to the complete disappearance of the contrast medium. Furthermore, liver can be completely scanned in the portal phase and delayed phase, and it is easier to detect small lesions in liver.^[24]^ With high SEN and accuracy, CEUS can be considered as a reliable method for follow-up after HCC. Many studies have confirmed that CEUS is of great value in predicting the recurrence after HCC. We performed a meta-analysis to determine the accuracy of CEUS in the prediction of the postoperative recurrence of HCC, so as to resolve the dispute.

## Author contributions

**Conceptualization:** Wencui Wu, Jieying Fu.

**Data collection:** Jia Tang.

**Funding acquisition:** Wencui Wu.

**Investigation:** Wencui Wu.

**Methodology:** Huan Luo.

**Project administration:** Wencui Wu.

**Resources:** Huan Luo, Jia Tang.

**Software:** Jia Tang, Huan Luo.

**Supervision:** Wencui Wu, Jia Tang.

**Validation:** Jia Tang.

**Visualization:** Jia Tang.

**Writing – original draft:** Wencui Wu, Jieying Fu.

**Writing – review & editing:** Wencui Wu, Jieying Fu.
